# Multidisciplinary Treatment of a Double First Mandibular Premolar 

**DOI:** 10.22037/iej.v12i4.17133

**Published:** 2017

**Authors:** Renato Piai Pereira, Rodrigo Ravazzi, Rogério Vieira Silva, Eduardo Nunes, João Milton Rocha Gusmão, Paulo Sérgio Flores Campos

**Affiliations:** a *Department of Health, Southwest Bahia University, Jequié, Bahia, Brazil; *; b * Private Practice, Vitória da Conquista, * *Bahia, Brazil*;; c *Department of Dentistry, Pontifical Catholic University of Minas Gerais, Belo Horizonte, Minas Gerais, Brazil;*; d *Department of Dentistry, Pontifical Catholic University of Minas Gerais, Belo Horizonte, Minas Gerais, Brazil; *; e *Department of Dentistry, Pontifical Catholic University of Minas Gerais, Belo Horizonte, Minas Gerais, Brazil; *; f * Department of Oral Radiology, School of Dentistry, Federal University of Bahia, Salvador, Bahia, Brazil*

**Keywords:** Double Tooth, Hemisection, Orthodontics, Root Canal Treatment

## Abstract

Gemination *aka* twinning and fusion, are rare occurrences in posterior mandibular teeth, often requiring endodontic and surgical treatment for functional, orthodontic or cosmetic reasons. The diagnosis and design of a precise treatment plan in cases involving double teeth are in most cases challenging. The purpose of this case report is to describe a successful multidisciplinary treatment protocol for a double tooth. Upon completion of the endodontic, restorative and orthodontic treatments, the clinical and radiographic three-year follow-up revealed that the rest of the transected premolar showed evidence of healing of the supporting tissues and satisfactory cosmetic result.

## Introduction

Tooth fusion is defined as the joining together of two or more adjacent teeth [1-5], and its prevalence in the permanent dentition is less than 1% [6-8]. The fusion process involves epithelial and mesenchymal germ layers, and results in an uneven dental morphology. It may occur between the dentin and/or enamel, in the pulp chamber and root canals, either connected or separately, depending on the stage of development when the joining occurred [9].

Moreover, gemination, *aka* twinning, is defined as an attempt made by the tooth germ to divide [1]. Twin teeth typically exhibit partially or fully joined pulp cavities, with roots normally joined together as one, with only a shallow groove in the area where the twins are connected [10].

It is clear, therefore, that in cases of fusion, the number of regular teeth is smaller, while in cases of twinning, the number of regular teeth is normal. However, when a regular tooth is fused with a supernumerary tooth, the differential diagnosis between fusion and gemination becomes challenging [3]. Therefore, cases of fused or geminated teeth have generally been called 'double teeth.'

The following case report describes a multidisciplinary treatment of a double lower left first premolar.

## Case Report

**Figure 1:**
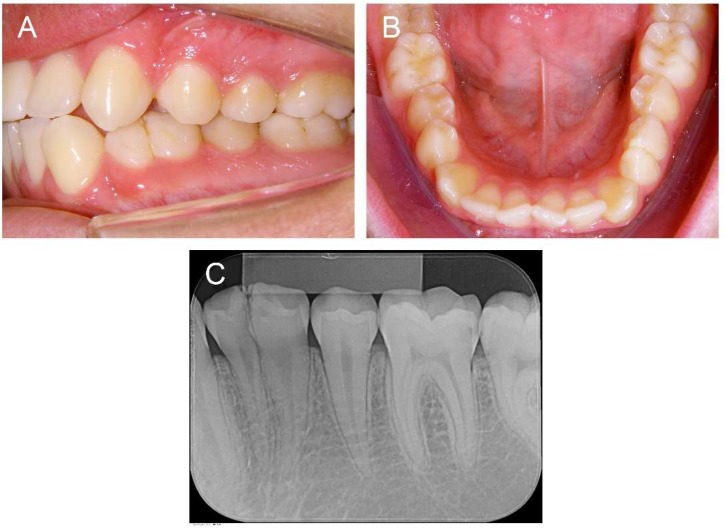
*Preoperative clinical examination of the double man* *dibular premolar:* A)* Left lateral view;* B)* Mandibular occlusal view;* C)* Periapical radiographic view*

The patient was a 20-year-old Caucasian man referred for dental treatment, whose chief complaint involved the straightening of misaligned teeth. The medical history revealed nothing relevant. The clinical examination showed a class II, division 1, right subdivision malocclusion, mild anteroinferior crowding, lower left canine in labioversion, mandibular midline shift and a double lower left premolar, which exhibited physiological mobility and a positive response to pulp sensitivity tests (Figure 1A to 1C).

The proposed orthodontic treatment aimed to achieve a class I relationship, correct the dental anteroinferior crowding and midline deviation. To these ends, the hemisection of the anomalous tooth was unavoidable.

**Figure 2:**
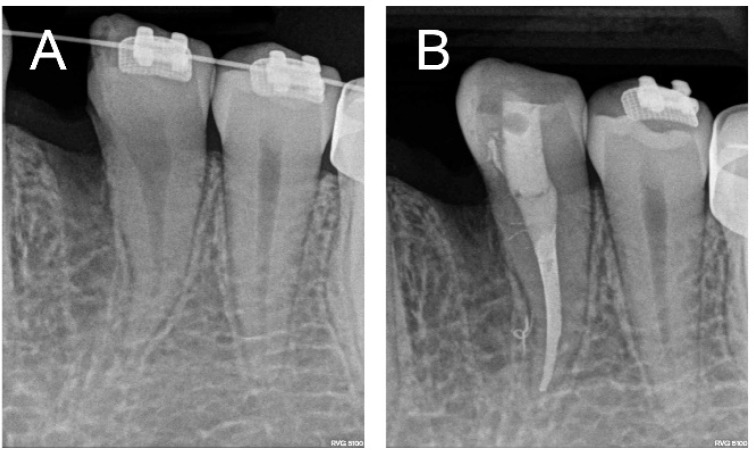
*Hemisection and extraction of the mesial portion of the premolar; *A)* Periapical radiograph before endodontic treatment; *B)* Periapical radiograph after endodontic treatment*

Thus, after placing a straight-wire fixed orthodontic appliance, the hemisection of the double tooth and subsequent extraction of its mesial half was carried out. The procedure was performed under local anesthesia by way of a mucoperiosteal flap and separation of the anterior segment of the tooth using a conic diamond bur under irrigation. The connection between the two coronary chambers was identified after removal of the mesial portion of the anomalous tooth, which determined the need for endodontic treatment (Figure 2A and 2B).

Endodontic treatment was performed in a single-visit. After local anesthesia the distal remaining portion of the tooth was isolated with a rubber dam. The endodontic access cavity was created with round and conic diamond burs. The working length was determined with a #15 K-type file coupled to an electronic apex locator (RomiApex A-15; Romidan, Kiryat Ono, Israel), and confirmed by radiographic examination. The root canals were instrumented manually with K-type files (Dentsply Maillefer, Ballaigues, Switzerland), by the crown-down technique under copious irrigation with 5.25% sodium hypochlorite (NaOCl) throughout the procedure, and 17% EDTA was used for smear layer removal. The canal was then suctioned, dried with paper points and obturated by using warm vertical compaction (Touch 'n Heat; Kerr, Orange, CA, USA), with gutta-percha and AH-Plus sealer (DeTrey/Dentsply, Konstanz, Germany).

**Figure 3:**
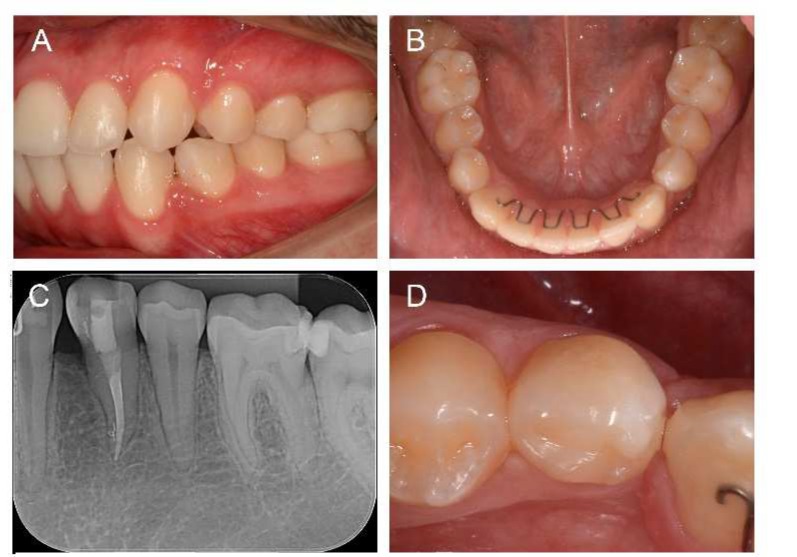
*Clinical examination after completion of orthodontic treatment, 3-year follow-up;* A)* Left lateral view;* B)* Mandibular occlusal view*; C)* Periapical radiographic view;* D)* Close-up view of premolar*

The restorative procedure was performed immediately following the endodontic treatment. The occlusal access cavity and mesial surface showed areas of dentine exposure due to hemisection and were restored to reestablish the original shape of a premolar. The clinical sequence proceeded with the application of 35% phosphoric acid gel (Ultradent, South Jordan, USA) for 20 sec. The enamel and dentin surfaces were washed and dried. The bonding agent (Ivoclar Vivadent, Schaan, Liechtenstein) was applied and light-cured according to the manufacturer's instructions. The nano-hybrid composite filling material for direct restorative procedures (IPS Empress Direct; Ivoclar Vivadent, Schaan, Liechtenstein) was inserted using incremental technique, with each increment being cured for 20 sec with LED curing light (Valo;Ultradent, South Jordan, UT, USA). After removing the rubber dam, the restoration was finished and occlusal adjustment was performed. 

The orthodontic treatment lasted approximately 36 months until the dental arches were coordinated and the occlusion was corrected. During this period a follow-up radiographic examination of the transected premolar was carried out (Figure 3A to 3D).

## Discussion

In view of the normal number of teeth in the dental arch, the case presented here could be diagnosed as tooth gemination. On the other hand, radiographs revealed the existence of two roots and two clearly discrete pulp cavities, a condition more frequently observed in cases of tooth fusion. Given this difficult diagnosis, we decided to adopt the terminology ‘double teeth,’ which can mean both gemination and fusion.

Double teeth are usually asymptomatic and, if aesthetically acceptable, do not require treatment. Whenever such condition causes aesthetic and functional problems such as dental caries in grooves, periodontal problems associated with grooves in fusion zones, endodontic complications, asymmetries and malocclusions [11], therapy is inevitable [12]. Treatment options include tooth extraction, hemisection and intentional replantation associated with endodontic and/or orthodontic treatment, crown reconstruction with fixed prostheses or composite resins [1-3, 8, 11, 13, 14].

Other more conservative, non-surgical approaches involve preserving the original form of the teeth, ensuring pulp health, selective endodontic treatment, mesiodistal crown reduction through stripping and restorative procedures [6, 9, 15-17].

Treatment of double teeth, however, depends on clinical condition and may require the input of a wide array of specialists, including orthodontists, endodontists, prosthodontists and oral surgeons in order to determine the best treatment approach possible with the most predictable outcome [2].

Once the need for orthodontic treatment had been established, this clinical case was planned to include surgery, probable endodontic therapy (confirmed during surgery), and restorative intervention. The point of root separation is an important determinant in the prognosis of double teeth. Teeth that are joined more apically cannot be separated without causing damage to most remaining and replanted teeth [11]. When the teeth are joined more coronally, hemisection is suggested to ensure smooth contours on the remaining tooth [11, 12].

In the case presented here, the hemisection was possible thanks to the complete separation of the dental roots, which proved to be an advantage since the success rates of reported intentional replantation run at 67% to 93% [11]. Failures are related to damage caused to the periodontal ligament, with the occurrence of root resorptions [13].

Communication between the coronary chambers of double teeth is a common phenomenon [9, 17]. In this case report, the evidence of communication between the pulp chambers was detected after hemisection and removal of the mesial portion. This determined the need for endodontic treatment.

## Conclusion

After a follow-up period of three years, the multidisciplinary approach advanced in this case revealed periapical and periodontal tissues within normal limits and provided satisfactory functional and aesthetic results.
